# On the Effects of 3D Printed Mold Material, Curing Temperature, and Duration on Polydimethylsiloxane (PDMS) Curing Characteristics for Lab-on-a-Chip Applications

**DOI:** 10.3390/mi16060684

**Published:** 2025-06-05

**Authors:** Rabia Mercimek, Ünal Akar, Gökmen Tamer Şanlı, Beyzanur Özogul, Süleyman Çelik, Omid Moradi, Morteza Ghorbani, Ali Koşar

**Affiliations:** 1 Faculty of Engineering and Natural Sciences, Sabanci University, Istanbul 34956, Turkey; rabia.mercimek@sabanciuniv.edu (R.M.); unalakar@sabanciuniv.edu (Ü.A.); gokmen.sanli@sabanciuniv.edu (G.T.Ş.); beyzanur.ozogul@sabanciuniv.edu (B.Ö.); omid.moradi@sabanciuniv.edu (O.M.); 2 Hydraulic Engineering, Civil Engineering and Geoscience, Delft University of Technology, 2628 CN Delft, The Netherlands; 3 Sabanci University Nanotechnology and Applications Center (SUNUM), Sabanci University, Istanbul 34956, Turkey; scelik@sabanciuniv.edu; 4 Center of Excellence for Functional Surfaces and Interfaces for Nano-Diagnostics (EFSUN), Sabanci University, Istanbul 34956, Turkey

**Keywords:** PDMS curing, organ-on-a-chip, 3D printing, Young’s modulus of elasticity, microfluidic devices

## Abstract

Soft lithography with microfabricated molds is a widely used manufacturing method. Recent advancements in 3D printing technologies have enabled microscale feature resolution, providing a promising alternative for mold fabrication. It is well established that the curing of PDMS is influenced by parameters such as temperature, time, and curing agent ratio. This study was conducted to address inconsistencies in PDMS curing observed when using different 3D-printed mold materials during the development of a Lab-on-a-Chip (LoC) system, which is typically employed for investigating the effect of hydrodynamic cavitation on blood clot disintegration. To evaluate the impact of mold material on PDMS curing behavior, PDMS was cast into molds made from polylactic acid (PLA), polyethylene terephthalate (PET), resin, and aluminum, and cured at controlled temperatures (55, 65, and 75 °C) for various durations (2, 6, and 12 h). Curing performance was assessed using Soxhlet extraction, Young’s modulus calculations derived from Atomic Force Microscopy (AFM), and complementary characterization methods. The results indicate that the mold material significantly affects PDMS curing kinetics due to differences in thermal conductivity and surface interactions. Notably, at 65 °C, PDMS cured in aluminum molds had a higher Young’s modulus (~1.84 MPa) compared to PLA (~1.23 MPa) and PET (~1.17 MPa), demonstrating that the mold material can be leveraged to tailor the mechanical properties. These effects were especially pronounced at lower curing temperatures, where PLA and PET molds offered better control over PDMS elasticity, making them suitable for applications requiring flexible LoC devices. Based on these findings, 3D-printed PLA molds show strong potential for PDMS-based microdevice fabrication.

## 1. Introduction

Lab-on-a-chip devices have emerged as promising platforms for many applications and the manipulation and control of minute volumes of fluids. These devices provide faster results, practical fabrication, a small footprint, higher efficiency, and lower cost compared to other methods among microfluidic platforms [1]. Within this realm, polydimethylsiloxane (PDMS) has become a material of choice for researchers and engineers and demonstrated remarkable mechanical flexibility, optical transparency, and compatibility with biological entities [2]. Its widespread adoption has propelled the development of diverse applications, varying from diagnostics [3] and biomedicine to environmental monitoring and drug delivery [4]. The advent of PDMS and soft lithography in the 1990s has triggered a transformative shift within the realm of microfluidics [5] and obviated the prerequisite for clean room infrastructure.

Regarding microfluidic devices, SU8 molds fabricated via microfabrication methods have been extensively used. However, due to their impracticality and high costs [6], more suitable alternative methods have been sought. Three-dimensional printing technologies have become a reliable alternative [7]. Especially in organ-on-a-chip devices used for complex multi-channel organs or tissue mimics, 3D-printed molds are increasingly used for rapid prototyping [8]. There are many studies where 3D printers not only fabricate molds but also microfluidic devices. For example, in the study of Musafargani et al. [9], the blood-brain barrier was modeled, and the microdevice designed for this model was fabricated with the help of a 3D printer. In the study of Alessandri et al. [10], a miniature device producing tiny, hollow sphere gels was fabricated. These spheres could hold cells and were coated inside with a thin layer of material that mimicked the natural environment surrounding cells in the body. Moreover, the recent work by Brivio et al. [11] further emphasizes the potential of microfluidic platforms for precision-engineered biochemical environments, supporting the growing relevance of customizable LoC systems.

Polydimethylsiloxane (PDMS) is a liquid prepolymer that becomes a flexible elastomer through a well-defined curing process. However, the intricate interactions of three critical parameters (temperature, curing agent ratio, and curing time) have a significant impact on this critical stage [1,12,13]. The temperature enhances the cross-linking rate, accelerating the cross-linking reaction between the PDMS base and curing agent. As the temperatures rise, the reaction rate increases, leading to faster curing times. However, it is essential to determine the ideal temperature balance to prevent unwanted side effects (bubbles, tackiness) [14,15]. Similarly, increasing the curing agent ratio results in stiffer PDMS, which improves the mechanical stability but at the cost of reduced flexibility, a trade-off depending on application needs [16]. In addition, the degree of cross-linking completeness is influenced by the curing time, with longer durations generally resulting in a more thoroughly cross-linked and mechanically stable polymer network. On the other hand, material deterioration might result from lengthy curing durations. In summary, the attainment of desired properties for diverse applications of PDMS curing necessitates meticulous adjustment of the temperature, curing agent ratio, and curing time.

While much attention has been given to PDMS formulation and curing conditions, comparatively little is known about the influence of mold material on the curing processes, especially in the context of 3D-printed molds. Many different materials can be used to fabricate molds for 3D printing types: resins (Stereolithography (SLA), Digital Light Processing (DLP), polylactic acid (PLA), polyethylene terephthalate (PET), polycarbonate (PC), and terephthalate (PETG). These differences can lead to favorable or adverse effects on PDMS curing. For instance, resins may inhibit curing due to residual photo initiators unless properly post-processed, while thermoplastics such as PLA and PET may support smoother curing at moderate temperatures but deform or warp under heavy heating conditions. The interaction between mold material and PDMS also affects the surface quality, cross-linking uniformity, and mechanical performance of the final device.

To the best of the authors, there is no study in the literature on the effect of different molds on PDMS curing. In this study, motivated by the lack of studies on this topic, resin, PET, PLA, and Al molds were used at different temperatures (55 °C, 65 °C, and 75 °C), and their curing characteristics were investigated for durations of 2, 6, and 12 h. Our findings aim to guide researchers in material selection and designing PDMS-based LoC devices using accessible 3D printing technologies.

## 2. Materials and Methods

Thermal properties of the mold material determine the curing time of PDMS and consequently the mechanical, thermal, and optical properties after curing. The widespread use of 3D printers has made the production of mold materials cheaper and facilitated the process. In related studies on microfluidic devices, a low margin of error is expected in fabrication. To address this issue, the effects of molds fabricated by a 3D printer and made from different materials (as well as aluminum with a high thermal conductivity) on the mechanical, thermal, and optical properties of PDMS were investigated. In this section, the materials used in this study, experimental setup, and mechanical, thermal, and optical characterization methods are covered.

### 2.1. Materials

PDMS (SYLGARD™ 186 Silicone Elastomer Kit, Dow Chemical Company, Michigan, United States) was used as the base elastomer due to its higher modulus and longer curing time compared to SYLGARD™ 184, which makes it more suitable for mechanical characterization. Sylgard 186 exhibits higher elongation at break, greater tear strength, and improved plasma bonding capability, making it more suitable for mechanically demanding applications requiring tunable stiffness, such as stretchable electronics and soft robotics [17]. Additionally, its enhanced mechanical robustness has been shown to improve the durability of flexible biomedical devices [18]. The mold materials included BASF Ultrafuse PLA Filament—Apricot Skin 2.85 mm, BASF Ultrafuse PET Filament—Transparent 2.85 mm, Formlabs Greyscale Resin, and Aluminum 7075.

### 2.2. Mold Design and Fabrication

**Figure 1:**
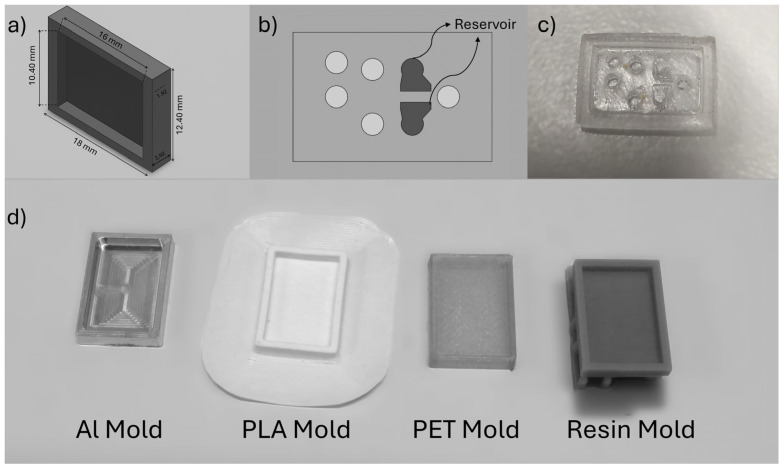
(**a**) Design of small-scale PDMS mold used in this study; (**b**) Representative design of a Lab on a Chip Device; (**c**) Real image of a LoC PDMS device; (**d**) Real images of molds fabricated with different materials.

Regarding the design, a configuration resembling a rectangular prism in the form of a simple closed chamber was considered to simulate PDMS-based microfluidic devices. The dimensions of this configuration are 1.92 mm in height, 10.4 mm in width, and 16 mm in length. Also, the molds have a wall thickness of 1 mm and a bottom surface thickness of 1 mm. These dimensions match those commonly used in microfluidic chips and allow for investigating PDMS curing characteristics. Figure 1a illustrates the dimensions of the molds used. As an example of a lab-on-a-chip application [19], Figure 1b,c show a device where PDMS served as a reservoir for blood clots. The system is a hybrid microfluidic platform composed of Si-glass and PDMS microchannels and was designed to investigate the effect of hydrodynamic cavitation on blood clot disintegration [19]. This study arose from a hypothesis developed after observing how different molds used in PDMS production influenced the curing process during our clot-on-a-chip research activity. To provide a more general contribution to the literature, a simplified mold design, without reservoirs or inlet/outlet holes, was used rather than replicating the original device geometry.

To prepare the mold configurations used in this study (Figure 1), 3D models were designed using the SOLIDWORKS 2021 software. Four different molds, PLA, PET, resin, and aluminum, were prepared using appropriate methods and equipment specific to each material. PLA molds were printed using an Ultimaker 3 3D printer (Ultimaker, Utrecht, Netherlands) (Fused Deposition Modeling, FDM) with the following parameters: nozzle diameter of 0.4 mm, nozzle temperature of 195 °C, bed temperature of 60 °C, 100% infill (grid pattern), layer height of 60 µm, and a print speed of 60 mm/s. Brim-type build plate adhesion was considered to ensure a flat base layer, with the cooling fan set to 100% after the first layer. No support was used. The slicing was performed using Ultimaker Cura 5.3.0. PET molds were fabricated using an Anycubic Kobra 2 (FDM) printer with a nozzle temperature of 215 °C, bed temperature of 70 °C, 100% infill, layer height of 150 µm, and a print speed of 50 mm/s. Similar to PLA, no supports were used, and brim-type build plate adhesion was performed. The cooling fan was set to100% after the first layer. The slicing was conducted using both the Anycubic proprietary slicer (fine detail profile) and Ultimaker Cura 5.3.0. Resin molds were printed using the Formlabs Form 3+ system (Formlabs, Somerville, MA, USA), which employed Low Force Stereolithography (LFS) technology. Standard Grey Resin v4 was used with a layer thickness of 50 µm. The laser system featured a 250 mW, 405 nm laser with an 85 µm spot size. Light exposure was automatically optimized by the LFS algorithm. The printing speed was 17 mm/h. Post-processing included washing in isopropanol (IPA) using the Form Wash system for 30 min, followed by post-curing under 405 nm UV light for 50 min using the Form Cure system. Aluminum molds were machined from Aluminum 7075 using a Mori Seiki NTX2000 9-axis (DMG Mori, Tokyo, Japan) CNC manufacturing platform, selected due to its high thermal conductivity, dimensional precision, and mechanical durability.

### 2.3. PDMS Curing

**Figure 2:**
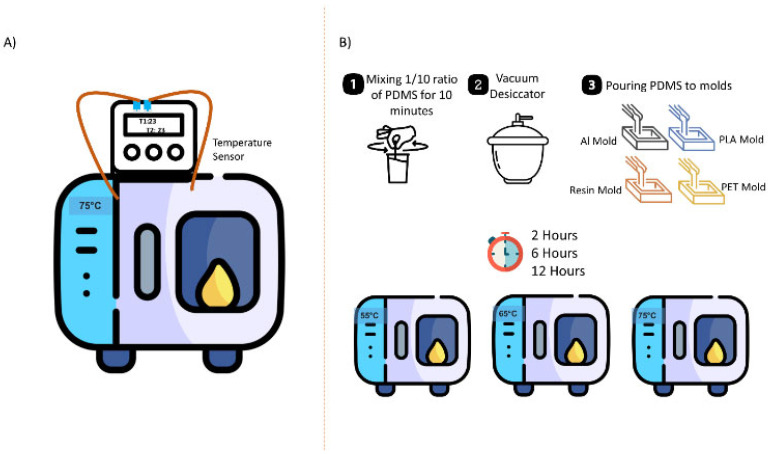
Experimental Setup: (**A**) Heat transfer experiment; (**B**) PDMS preparation and curing process.

For PDMS chip fabrication, a mixture of PDMS elastomer base and curing agent with a ratio of 10:1 was stirred for 10 min and then poured into molds after vacuuming to remove all bubbles. The mixture was cured at different temperatures (55 °C, 65 °C, and 75 °C) in a preheated oven (Binder, Tuttlingen, Germany). Additionally, three different curing times, namely 2 h, 6 h, and 12 h, were considered (Figure 2). The PDMS samples obtained from these experiments were then evaluated in terms of mechanical, thermal, chemical, and morphological features.

### 2.4. Heat Transfer Tests

Polydimethylsiloxane (PDMS) has been extensively used due to its advantageous properties, such as low density, high flexibility, ease of molding, and relative biocompatibility. Pure PDMS has a thermal conductivity of only 0.16 W/mK at room temperature [20,21]. Polymers generally have low thermal conductivity, which is lower than 0.5 W/mK compared to metals [22].

The conduction heat transfer rate between molds and the PDMS is expressed as:

Q_cond_ = kA ΔT/d (1)

where Q represents the heat transfer rate measured in Watts, k denotes the thermal conductivity of the material quantified in Watts per meter per Kelvin, A is the cross-sectional area through which the heat is transmitted measured in square meters, ΔT indicates the temperature differential across the materials expressed in Kelvin, and d represents the thickness of the material measured in meters [23,24]. The thermal conductivities associated with the various materials employed in this study are meticulously documented in Table 1.

To facilitate the acquisition of precise temperature data, a k-type thermocouple was strategically positioned on the bottom surface of each of the molds, allowing for accurate readings during the experiment (Figure S1). Then, PDMS was poured on it, and it was kept between the PDMS and the mold. Subsequently, the molds were placed simultaneously into an oven that had been rigorously preheated to the specified temperature of 75 °C, a temperature that was deliberately chosen as the standard environment for the curing process of PDMS.

### 2.5. Characterization Methods

Multiple techniques were implemented for characterization. First, the elasticity of 36 different PDMS samples was analyzed by atomic force microscopy (AFM) to analyze the surface roughness of the molds and to calculate the Young’s modulus of elasticity of the PDMS samples. Soxhlet analysis was employed to determine the curing rates. Thermogravimetric analysis (TGA) was performed to provide insights into the thermal stability of the samples. Additionally, Fourier-transform infrared spectroscopy (FTIR) was performed to compare PDMS samples cured in different molds. Finally, scanning electron microscopy (SEM) revealed the surface morphology of the PDMS samples.

#### 2.5.1. Atomic Force Microscopy (AFM) Characterizations

Surface roughness analysis based on AFM was made to further evaluate the interaction between the mold surface and PDMS. AFM measurements were performed using a Bruker Multimode 8 system. A silicon cantilever with a spring constant of 30 N/m was used in tapping mode. The oscillation frequency ranged from 1 to 10 kHz, depending on the characteristics of the sample surface. The choice of oscillation frequency was primarily influenced by the nature of the tip–sample interaction. Since the tapping mode was employed, the instrument’s automatic force optimization feature was active throughout the measurements. This function helped to regulate the applied force to minimize potential damage to the tip. As a result of this automated adjustment, the oscillation frequency varied according to the mechanical properties of each sample, generally shifting depending on whether the sample was soft or hard.

The sample holder accommodates samples within a circular area of 2 cm in diameter, and proper placement within this area is essential to enable *Z*-axis movement during scanning. The utilized piezo scanner was the AS-130 (“J”) head, which provides a nominal scan area of 125 µm × 125 µm in the lateral (XY) direction and a 5.0 µm vertical (Z) range.

The measurements were repeated on five distinct regions for each mold type, using the XY offset function (lateral movement) of the AFM stage to deliberately avoid areas with residual PDMS. This ensured that the scans represented clean and consistent surface areas of the mold materials. The obtained results are presented in Table 2. All roughness measurements were made using a scan size below 50 µm × 50 µm to minimize image distortion, especially for soft surfaces, and leveling/post-processing steps were applied consistently.

For locally measuring the Young’s modulus, a silicon nitride cantilever with a nominal spring constant of ~0.3 N/m was employed using AFM with nanoscale quantitative mechanical measurement capability. The applied loading force was approximately 2 µN, and the indentation depth was maintained in the range of 1.0–1.2 µm, with a loading rate of 1 µm/s to minimize time-dependent effects.

#### 2.5.2. Soxhlet Analysis

PDMS-cured samples were characterized by Soxhlet extraction (Figure S1). In the study of Lee et al. [29], 38 different solvents were tested for PDMS, and chloroform was selected as one of the fifth-best solvents so that chloroform was used to dissolve uncured PDMS. Accordingly, each specimen (approximately 0.3–0.4 g,) was extracted with chloroform for 24 h and dried in a 70 °C oven for 24 h. After drying, the samples were weighed one by one, and the curing percentage was determined.

% Curing = (m_2_/m_1_)100 (2)

where m_1_ is the mass of PDMS cured before extraction, and m_2_ is the mass of cured PDMS after extraction.

#### 2.5.3. Thermogravimetric Analysis

The thermal stability and degradation characteristics of the cured PDMS samples were systematically examined utilizing TGA (Shimadzu DTG-60H, Shimadzu Corporation, Kyoto, Japan), which was operated at temperatures reaching 1000 °C with a heating rate of 10 °C per minute and conducted under a controlled nitrogen atmosphere.

#### 2.5.4. Fourier Transform Infrared Spectroscopy Analysis

Fourier Transform Infrared Spectroscopy analysis was performed with a FTIR (Thermo Scientific/IS10, Thermo Fisher Scientific, Waltham, MA, USA) device by taking 64 scans in the range of 400–4000 cm^−1^ for all the cured samples.

#### 2.5.5. Scanning Electron Microscopy (SEM) Analysis

The surface morphologies of the samples were examined by a field emission scanning electron microscope (FESEM, Leo Supra 55VP, ZEISS, Jena, German).

## 3. Results

### 3.1. Atomic Force Microscopy (AFM) Results

#### 3.1.1. Surface Roughness Analysis of Prepared Molds

**Figure 3:**
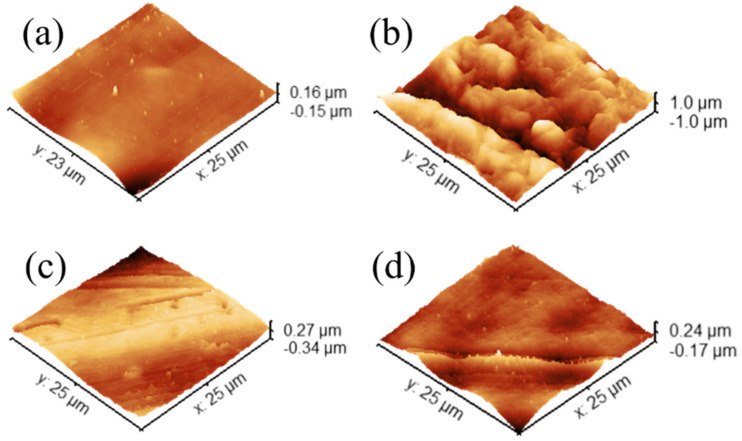
Three-dimensional AFM images of mold surfaces in contact with PDMS (**a**) PLA, (**b**) PET, (**c**) Al, and (**d**) resin mold, respectively.

3D and 2D scan results are presented in Figure 3 and Figure S2, respectively. For the evaluation of roughness, two commonly reported parameters are considered here to assess the surface roughness: arithmetic mean roughness (Sa) and the root mean square roughness (Sq) [30,31]. Roughness calculations were performed using the Gwyddion© software’s statistical analysis tool. Both Sa and Sq can be used to quantify the surface deviation from the reference plane (often the mean height).

Sa is the arithmetic mean of the absolute deviations of the surface height from the mean plane. In this case, the smoothest and homogenous surface belongs to molds. Mathematically, if the height z (x, y) is considered as a surface function dependent on given coordination on the surface, the mean height $\bar{z}$ becomes:

(3) $$S_{a}=\frac{1}{A}\iint \left|z\left(x,y\right)-\bar{z}\right|dxdy$$

where A is the analyzed area.

Sa provides a direct, linear measurement of the average surface deviation. It gives the conventional roughness level of the surface. However, Sa does not weigh large peaks or deep valleys more heavily than smaller features. Each deviation magnitude is taken at face value and then averaged.

On the other hand, Sq is the square root of the mean of the squared height deviations and is expressed as:

(4) $$S_{q}=\sqrt{\frac{1}{A}\iint {\left(z\left(x,y\right)-\bar{z}\right)}^{2}dxdy}$$

where A is the analyzed area.

Since Sq squares the height deviations before averaging, larger deviations are more emphasized with this parameter. Few high peaks or deep pits will disproportionately increase the Sq value, which makes Sq more sensitive to surface outliers or large topographical features. The comparison of Sq and Sa ratios presents critical interpretive guidance over the obtained data. For Sq > Sa, there are notable peaks or valleys that dominate the surface profile. In other words, the surface has likely a non-Gaussian height distribution with more pronounced extremes. For Sq ≈ Sa, the surface topography is more uniformly distributed, with fewer extreme features, and there is a more even (or near-Gaussian) roughness distribution.

As can be seen from the data in Table 2, for PLA (Figure 3a), the difference between RMS and mean roughness is significant (≈1.58). While the absolute roughness is relatively low, the ratio suggests that there are notable peaks or valleys making the surface spikier compared to a surface where Sq and Sa are closer to each other. These spikier localized regions are remnant PDMS particles that adhere to the surface of the mold. This conclusion arises from the inphase mapping image (Figure S3) which indicates the presence of more elastic regions appearing both in the height and in-phase images. The negative values of the inphase map image indicate that the cantilever responds in a delayed manner to the drive signal and suggest higher energy dissipation, typically associated with softer, adhesive, or more viscous materials.

On the other hand, the PET surface has the highest overall roughness among the samples. The surface is very rough compared to the others. However, the ratio of Sq to Sa is closer to 1 (≈1.21), indicating that although the surface is rough, the distribution is uniform. The peaks and valleys do not dominate disproportionately, and the entire surface is rather consistently irregular at a high scale. The aluminum surface is moderately rough. Its Sq and Sa values are relatively close to 1 (≈1.2), suggesting a more uniform distribution of topographical features without extremely pronounced peaks or pits. The overall roughness of the resin surface is intermediate (≈1.31). Its ratio is higher than PET and Al but lower than PLA. This implies that the resin surface has some irregularities and few pronounced features, but not to the same degree as PLA. Overall, the PET surface stands out with the highest overall roughness but relatively uniform features. The PLA surface is the smoothest one but displays a larger difference between Sq and Sa, indicating more pronounced localized surface features. Although the Al surface is rougher than PLA and resin surfaces, its surface is more even in terms of the feature distribution. Al and resin surfaces have a moderate roughness and a moderately uniform distribution of surface features.

#### 3.1.2. Local Young’s Modulus of Elasticity Measurements on Obtained PDMS Samples

The Young’s modulus of elasticity is the most crucial mechanical property to consider for PDMS samples. It defines the linear relationship between the unit strain and normal stress (tensile or compressive) during elastic deformation. Essentially, it is stress divided by strain. While tensile tests are commonly used to measure it, the small size and highly elastic nature of PDMS samples make obtaining reliable results challenging. Therefore, the AFM method was employed to obtain more precise measurements on the tiny PDMS samples.

To calculate the Young’s modulus of elasticity using AFM, it is necessary to use the tapping mode [32]. In this mode, the cantilever gently applies and withdraws slight pressure on the sample, i.e., it performs a gentle tapping and retraction movement. This movement resembles the motion of a flexible spring, and an energy transformation occurs at the point of contact:

ΔE_p,s_ = 1/2 k_s_ (A_0_^2^ − A^2^) (5)

where A_0_ and A are the free and restricted amplitudes of the cantilever, respectively [33].

The spring constant (k_s_) of the sample is related to the energy stored in it (ΔE_p,s_) at each cantilever contact [34]:

ΔE_p,s_ = 1/2 k_s_ (h_0_ − h)^2^ (6)

The Hertzian model yields the following formula, where the tip can be regarded as a sphere of radius R with a significantly greater Young’s modulus of elasticity than that of the flat sample:

(h_0_ − h)^3^ = [9 (1 − v)^2^/16 RE] ^2^ F^2^ (7)

Here, F is the loaded force, E is the Young’s modulus of elasticity, w_s_ is the width measured at half-maximum, and v is the Poisson ratio [35]. The following formula can be used to obtain the Young’s modulus of elasticity, assuming a normal Poisson ratio of 1/3 [36]:

E = F/w_s_ h^1/2^ (h_0_ − h)^−3/2^ (8)

It is important to acknowledge the limitations of the Hertz model when applied to PDMS. Since PDMS is known to be viscoelastic and time-dependent, deviations from ideal elastic behavior are expected. The assumption of purely elastic, isotropic behavior introduces approximation errors, particularly at longer indentation times or higher loads. Nonetheless, due to the small loading rate and shallow indentation depths used in this study, the Hertz model remains a reasonable approximation for comparing relative stiffness across samples under consistent conditions.

**Figure 4:**
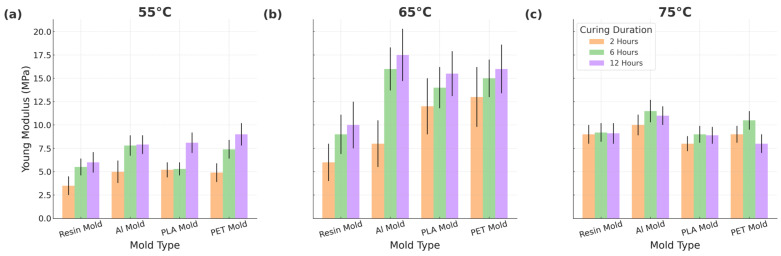
Young’s modulus elasticity of PDMS samples cured in different molds at different times and temperatures (**a**) at 55 °C, (**b**) at 65 °C and (**c**) at 75 °C.

The results of Young’s modulus of elasticity are displayed in Figure 4. The Young’s modulus of elasticity values of PDMS cured at the lowest temperature (55 °C) are lower than those cured at higher temperatures, as expected due to the reduced curing ratio. However, the Young’s modulus of the elasticity of samples cured at 65 °C unexpectedly surpasses those cured at 75 °C. This observation shows that there is no directly proportional relationship between the curing temperature and Young’s modulus of elasticity. Interestingly, our study reveals that while the different mold materials do not significantly impact the overall curing, their inherent thermal properties influence the PDMS curing reaction kinetics. This implies that rapid or slow curing can potentially affect the mechanical properties of the final PDMS product.

While the mold material has no significant effect on the curing efficiency (as confirmed by Soxhlet and thermal analysis), differences in the thermal conductivity among mold types influence the curing rate, which in turn affects mechanical outcomes. These results show that the choice of the mold material can alter the local curing dynamics, indirectly impacting the final elastic properties of PDMS.

### 3.2. Soxhlet Curing Analysis

**Figure 5:**
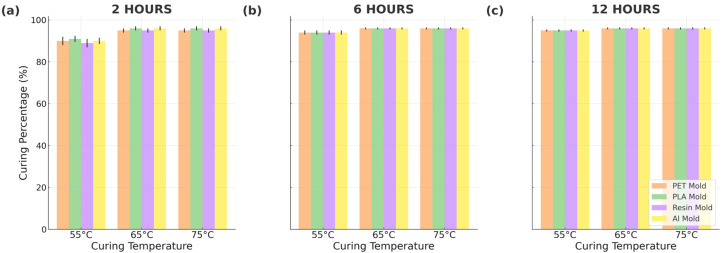
Soxhlet analysis results for different curing times: (**a**) 2 h, (**b**) 6 h, and (**c**) 12 h.

The results of Soxhlet curing analysis are presented in Figure 5. As can be seen, the type of mold used in the fabrication process has a negligible effect on the overall curing efficiency of PDMS. Notably, only the samples cured at a lower temperature (55 °C) exhibit a significantly lower degree of crosslinking for a shorter duration (2 h) compared to all other samples. This suggests that the curing temperature and time have a more pronounced effect on the crosslinking process than the specific mold type.

### 3.3. Heat Transfer Experiments

**Figure 6:**
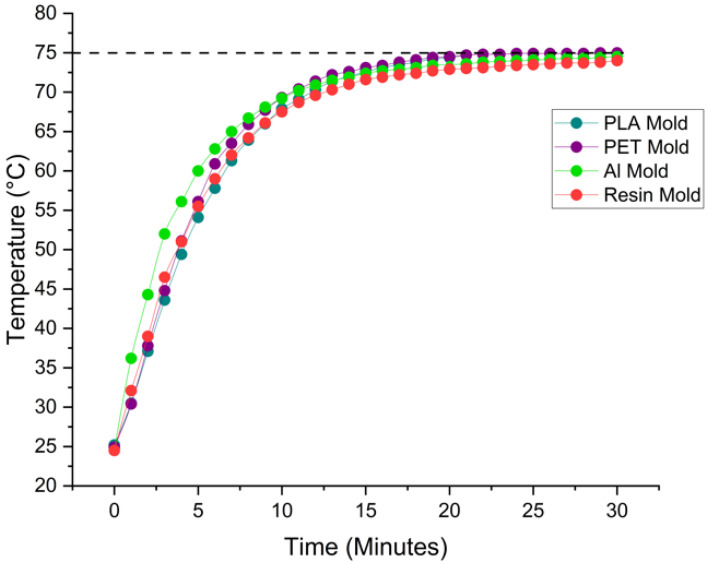
Average surface temperature profiles of molds during PDMS curing at 75 °C, recorded over a 30-min interval.

Heat transfer experiments were conducted at a set oven temperature of 75 °C over a period of 30 min, with each trial repeated three times to ensure data consistency and reliability. Figure 6 presents the average temperature profiles of the different mold materials, illustrating their thermal response over time during PDMS curing. As expected, the aluminum mold—known for its high thermal conductivity—reaches the target temperature significantly faster (within approximately 19 min). PLA and PET molds exhibit similar thermal behavior, reaching equilibrium around 25 min. The resin mold, however, has a distinctly slower thermal response.

The apparent discrepancy between Figure 6 and Table 3 arises from the difference in measurement interpretation. Figure 6 shows the average surface temperature trends across the molds, while Table 3 specifically highlights the total time required for the internal temperature of each mold to uniformly reach 75 °C, particularly at the geometric center of the mold where heat penetration is slowest. For resin, due to its lower thermal conductivity and potential insulating behavior, the internal temperature continues to lag even after the surface approaches the oven setpoint—leading to the >40 min value listed in Table 3. This difference emphasizes the importance of distinguishing between the surface temperature equilibration and full internal thermal stabilization, particularly when working with low-conductivity materials such as resin.

### 3.4. Thermogravimetric Analysis Results

**Figure 7:**
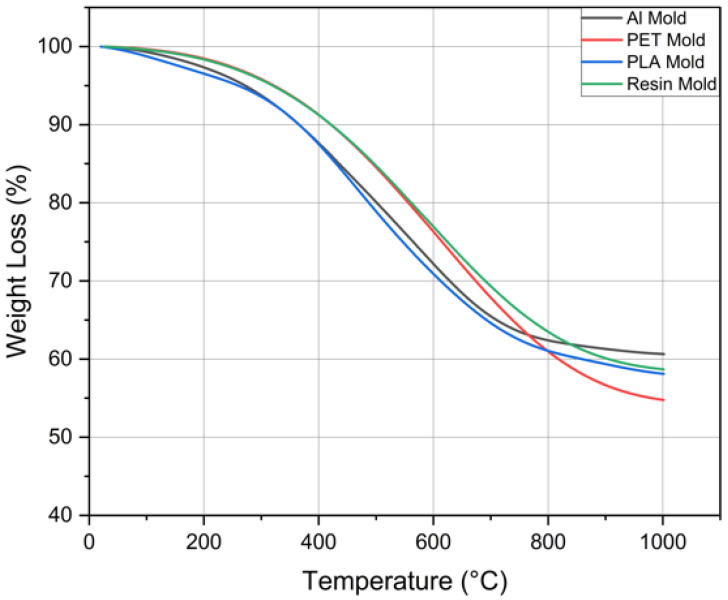
TGA analysis of PDMS samples.

TGA analyses were performed for 4 different PDMS samples for a shorter duration (2 h) prepared in different molds at 55 °C. The results of TGA analysis are shown in Figure 7. Accordingly, the starting temperature of degradation is around 205 °C. As a result of the Soxhlet analysis, although the curing percentages are almost the same at 55 °C for 2 h, the mass loss is found to be less for Al molds when the mass loss is examined in TGA. While the mass loss of PDMS is 38% when an Al mold is used, the mass loss is 42% for PDMS cured in PLA or resin molds and 45% for PDMS cured in PET mold. The thermal conductivity and mass loss of PLA, resin, and PET molds are close to each other. The thermal conductivity of the aluminum mold is considerably higher than the other molds, which results in less mass loss than the others. While this is initially attributed to more uniform thermal curing, it is also plausible that the chemical inertness of aluminum surfaces reduces unwanted side reactions or degradation during the curing process. This suggests that the mold surface chemistry may also contribute to the thermal stability, in addition to the heat transfer characteristics. The PLA, resin, and PET molds do not have a perfectly homogeneous cure, which causes a difference in the TGA results.

### 3.5. FTIR Analysis Results

**Figure 8:**
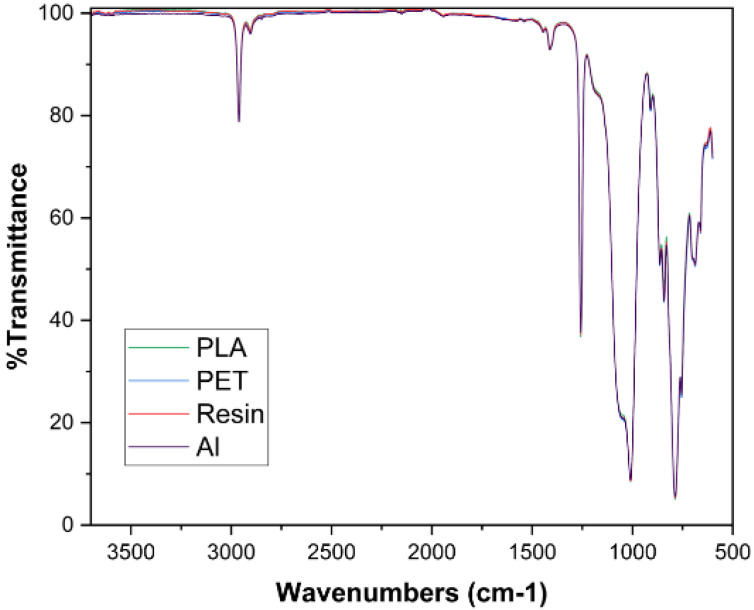
FTIR results of PDMS samples.

Figure 8 displays the FTIR spectra of the cured PDMS samples. The results were obtained from the sides of samples that were in contact with the mold. The spectra profile and peak position of all samples overlapped and remained intact. This is due to the homogeneous curing in PDMS samples, which led to coherent chemical uniformity in all samples as there is no change in functional groups during curing in different molds. The asymmetric stretching of CH_3_ in the Si-CH_3_ bond is observed around 2960 cm^−1^ wavelength. There is a bending motion of C-H molecules in methyl groups around 1410 cm^−1^. At 1260 cm^−1^, symmetric deformation motions of CH_3_ in the Si-CH_3_ bond can be seen. Si-O-Si bonding is observed around a wavelength of 1000–1050 cm^−1^. The wavelength of 755–790 cm^−1^ indicates the rocking of Si-C in the Si-CH_3_ bond.

### 3.6. Scanning Electron Microscopy Images

**Figure 9:**
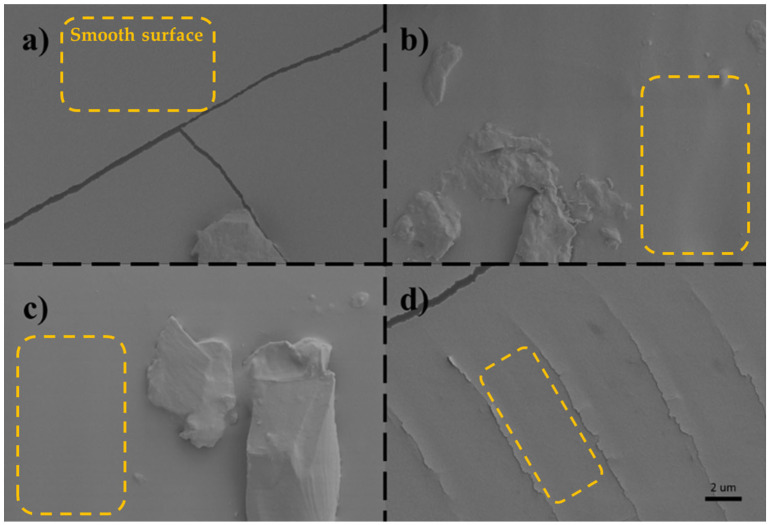
SEM images of PDMS samples cured with (**a**) PLA, (**b**) PET, (**c**) Al and (**d**) resin mold, respectively. Smooth surfaces of PDMS samples are highlighted in dashed squares.

The scanning electron microscopy (SEM) image (Figure 9) reveals the surface morphology of the PDMS samples. While the overall surfaces appear to be smooth, a close observation discloses the presence of localized roughness. It was aimed to include crack regions with rough surface features in the SEM images to enhance contrast and to improve visual clarity. On flat and smooth surfaces, SEM images often appear uniformly gray, lacking sufficient contrast to distinguish the surface details. By highlighting the areas surrounding the cracks, where topographical variation is more pronounced, it was sought to make the images more interpretable to readers.

This surface imperfection is attributed to the inherent roughness of the mold utilized in the fabrication process. As PDMS readily conforms to the mold topography, any imperfection on the mold is replicated on the PDMS surface. Consequently, achieving a pristine and smooth PDMS surface necessitates heightened attention to both the resolution of the 3D printed mold and the meticulous removal of the PDMS during demolding. Aside from cracks and some local inhomogeneous areas on the surface, comparative analysis of molds, SEM images, and observations indicate that PDMS samples fabricated using PLA molds exhibit smoother surface morphology, in contrast to those fabricated with other molds, which display a higher degree of surface roughness. This discrepancy can be attributed to variations in the manufacturing resolution of molds, as previously discussed.

## 4. Discussion

According to our findings, curing at lower temperatures is more controllable with PLA and PET molds, making them suitable for tuning the elasticity of PDMS-based microfluidic chips in Lab-on-a-Chip applications. However, these materials begin to deform or lose dimensional stability at elevated temperatures above approximately 70–80 °C, limiting their suitability for high-temperature curing processes. On the other hand, the utilization of a resin mold introduces challenges depending on the specific resin employed, as chemical reactions might occur. This issue is elucidated in the investigation conducted by Bazaz et al. [37], wherein it was demonstrated that the use of DPL resin was not conducive to the curing of PDMS. To address this concern, potential remedies include altering the resin type or implementing post-3D printing treatments on the resin mold [37]. It is noteworthy to underscore that microscale fabrication of aluminum (Al) mold-based devices is inherently more intricate and cost-intensive when compared with 3D printing methodologies. However, the incorporation of aluminum mold in this study is justified by its exceptional heat conductivity properties. As the mold size decreases, the surface area directly exposed to heat from the oven to the PDMS mixture decreases, which emphasizes the significance of mold wall thickness and material properties [38]. In smaller dimensions, the heat dissipated into the mold during curing might result in non-uniform distribution due to the modest thermophysical properties of the mold, influencing the mechanical properties of PDMS accordingly [39]. On the other hand, in larger dimensions, where the surface area is large, the material properties of the mold could be disregarded [40].

## 5. Conclusions

This study examined the influence of different mold materials—PLA, PET, resin, and aluminum—on the curing behavior and mechanical properties of PDMS for Lab-on-a-Chip (LoC) applications. According to the results, while the overall curing efficiency, as assessed by Soxhlet extraction and TGA, remains relatively consistent across mold materials under the selected temperature and time conditions, the mechanical properties, particularly Young’s modulus, are affected by both the mold material and curing conditions.

Mechanical characterization revealed that higher curing temperatures and longer durations promote a more crosslinked PDMS network, leading to increased stiffness. Among the mold materials, aluminum molds yielded PDMS samples with the highest Young’s modulus (~1.84 MPa at 65 °C), while PLA and PET produced softer samples, suggesting their suitability for applications requiring more flexible substrates. Although thermal ramp profiles across molds were not dramatically different, minor variations, likely due to differences in the thermal conductivity, may contribute to localized differences in the cross-linking behavior.

Surface characterization through SEM and AFM further revealed that PDMS samples cast from PLA molds exhibited smoother and more uniform surfaces, likely due to favorable mold-PDMS interactions and low chemical reactivity. These properties are advantageous for applications where surface fidelity and optical clarity are critical.

Rather than claiming a universally superior mold material, this study provides guidance for selecting 3D-printed molds based on specific application needs. Accordingly, aluminum molds are more suitable for rigid, mechanically robust microfluidic components, while PLA molds offer smoother PDMS surfaces and adequate elasticity for applications requiring flexible, optically clear microchannels, such as wearable sensors or organ-on-chip platforms. Resin molds may require additional post-processing to avoid potential curing inhibition.

By presenting a systematic comparison, this study will contribute to more informed material selection in the design and fabrication of PDMS-based microfluidic devices. These findings can guide future research efforts focusing on tuning PDMS properties for application-specific LoC systems, especially in prototyping workflows using accessible 3D printing technologies.

## Figures and Tables

**Table 1:** Thermal conductivities of mold materials.

Mold Material	Thermal Conductivity (at 25 °C)	Reference
Al	126 W/mK	[25]
PET	0.24 W/mK	[26]
PLA	0.13 W/mK	[27]
Resin	0.28 W/mK	[28]

**Table 2:** Arithmetic roughness values (Sq) and mean roughness (Sa) values obtained from molds using AFM analysis.

Mold Type	Roughness Values		
	Arithmetic Roughness (Sq) ± SD	Mean Roughness (Sa) ± SD	Sq/Sa
PLA	22.42 ± 2.8 nm	14.23 ± 1.9 nm	≈1.58
PET	364.57 ± 20.5 nm	297.84 ± 14.6 nm	≈1.21
Al	98.30 ± 7.2 nm	81.89 ± 5.3 nm	≈1.20
Resin	36.63 ± 4.4 nm	28.02 ± 2.6 nm	≈1.31

**Table 3:** Time required for each mold type to achieve thermal equilibrium (uniform 75 °C) at the core of the mold.

Mold Type	Reaching Time to Oven Temperature (75 °C)
PLA	25 min
PET	25 min
Al	19 min
Resin	Over 40 min

## Data Availability

Data will be made available on request.

## Supplementary Materials

The following supporting information can be downloaded at: https://www.mdpi.com/article/10.3390/mi16060684/s1, Figure S1: Experimental schematic of (a) Heat transfer tests, (b) Soxhlet analysis.; Figure S2: 2D AFM images of mold surface in contact with PDMS (a) PLA, (b) PET, (c) Al and (d) Resin molds, respectively (Scale bar = 5 mm); Figure S3: (a) 2D AFM height images of mold surface in contact with PDMS (b) Corresponding linear profile for height map (c) Inphase map of scanned area and (d) Corresponding linear profile for inphase map (Scale bar = 5 mm).

## Abbreviations

The following abbreviations are used in this manuscript:

PDMS	Polydimethylsiloxane
PET	Polyethylene terephthalate
PLA	Polylactic acid
Al	Aluminum
SEM	Scanning Electron Microscopy
TGA	Thermogravimetric Analysis
FTIR	Fourier transform infrared (FTIR) spectroscopy
AFM	Atomic Force Microscopy
LoC	Lab-on-Chip
