# Supplementary material for: On the Effects of 3D Printed Mold Material, Curing Temperature, and Duration on Polydimethylsiloxane (PDMS) Curing Characteristics for Lab-on-a-Chip Applications
Source: Micromachines (Basel). 2025 Jun 5;16(6):684. doi: 10.3390/mi16060684 (PMC12195046; doi:10.3390/mi16060684)
Supplement: Supplementary file 1 [file micromachines-16-00684-s001.zip › micromachines-3648280-supplementary.pdf]

Supplementary Materials

On the effects of 3D Printed Mold Material, Curing Temperature, and Duration on Polydimethylsiloxane (PDMS) Curing Characteristics for Lab-on-a-Chip Applications

Rabia Mercimek [1], Ünal Akar [1], Gökmen Tamer Şanlı[2], Beyzanur Özogul[1], Süleyman Çelik[3], Omid Moradi[1,4], Morteza Ghorbani[1,3,4] * and Ali Koşar [1,3,4] *.

[1] Faculty of Engineering and Natural Sciences, Sabanci University, 34956 Tuzla, Istanbul, Turkey.
[2] Affiliation 2; e-mail@e-mail.com

* Corresponding author, E-mails: kosara@sabanciuniv.edu, morteza.ghorbani@sabanciuniv.edu

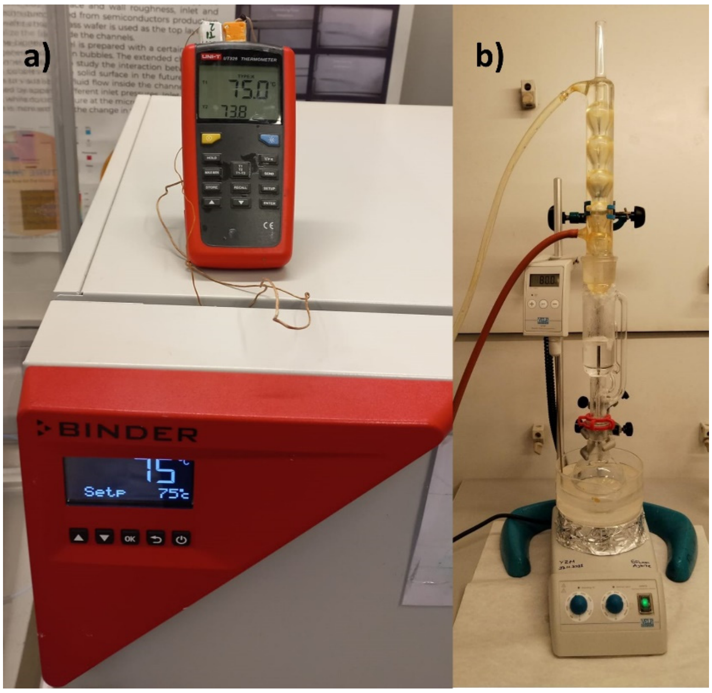

Figure S1: Experimental schematic of a) Heat transfer tests, b) Soxhlet analysis.

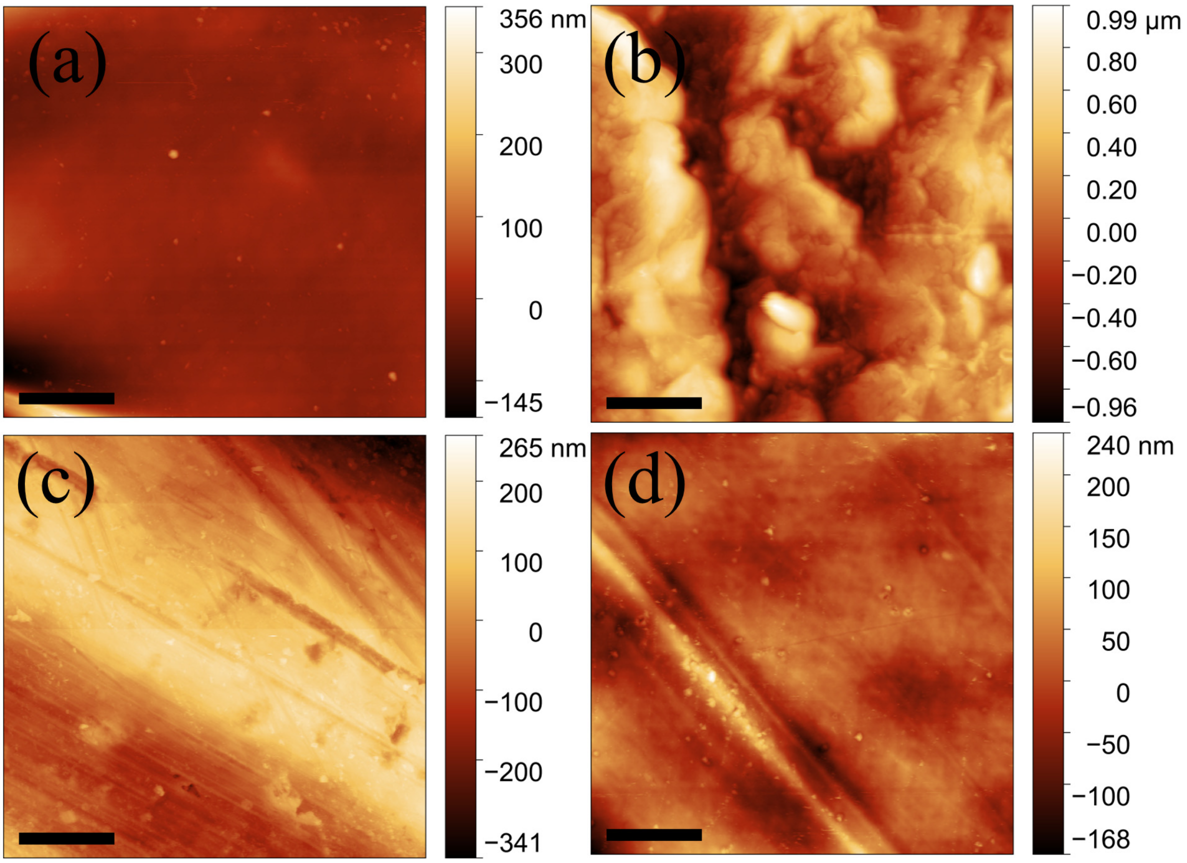

Figure S2: 2D AFM images of mold surface in contact with PDMS a) PLA, b) PET, c) Al and d) Resin molds, respectively (Scale bar=5µm).

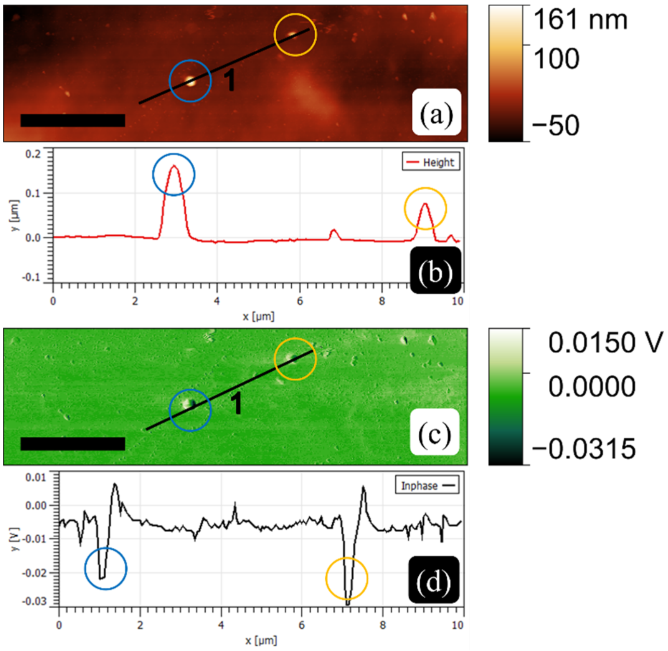

Figure S3: a) 2D AFM height images of mold surface in contact with PDMS b) Corresponding linear profile for height map c) Inphase map of scanned area and d) Corresponding linear profile for inphase map (Scale bar=5μm).
